# Age-Related Macular Degeneration Revisited: From Pathology and Cellular Stress to Potential Therapies

**DOI:** 10.3389/fcell.2020.612812

**Published:** 2021-01-25

**Authors:** Majda Hadziahmetovic, Goldis Malek

**Affiliations:** ^1^Duke Eye Center, Department of Ophthalmology, Duke University School of Medicine, Durham, NC, United States; ^2^Department of Pathology, Duke University School of Medicine, Durham, NC, United States

**Keywords:** age-related macular degeneration, oxidative stress, retinal pigment epithelial (RPE), choroidal endothelial cells, therapy

## Abstract

Age-related macular degeneration (AMD) is a neurodegenerative disease of the aging retina, in which patients experience severe vision loss. Therapies available to patients are limited and are only effective in a sub-population of patients. Future comprehensive clinical care depends on identifying new therapeutic targets and adopting a multi-therapeutic approach. With this goal in mind, this review examines the fundamental concepts underlying the development and progression of AMD and re-evaluates the pathogenic pathways associated with the disease, focusing on the impact of injury at the cellular level, with the understanding that critical assessment of the literature may help pave the way to identifying disease-relevant targets. During this process, we elaborate on responses of AMD vulnerable cells, including photoreceptors, retinal pigment epithelial cells, microglia, and choroidal endothelial cells, based on *in vitro* and *in vivo* studies, to select stressful agents, and discuss current therapeutic developments in the field, targeting different aspects of AMD pathobiology.

## Introduction

Age-related macular degeneration (AMD) is the leading cause of irreversible central vision loss in the Western hemisphere (Wong et al., 2014). It has been postulated that with the growing aging population, the prevalence and burden of AMD will continue to rise. In the early stages of the disease, visual deficits include impaired dark adaption, but otherwise may be minimal. However, as the disease evolves, vision becomes progressively more compromised, the retinal tissue degenerates, and suffers permanent damage. Traditionally, AMD has been classified broadly into two clinical sub-types; dry or non-exudative and wet/neovascular or exudative (Ferris et al., 2013; Spaide et al., 2018). In developed countries, approximately 10% of the population over the age of 65 years and 25% over the age of 75 years are purported to have been diagnosed with AMD. It is further estimated that in the US, about 11 million people (∼85% of all AMD) have dry AMD, while 1.5 million (∼15% of all AMD) are affected by the advanced stages of the disease (Joachim et al., 2015; Chou et al., 2016), with an estimated 70,000 new cases of wet AMD identified each year (Rudnicka et al., 2015). Though select treatment options targeting vascular leakage and stability are available for patients presenting with the wet or neovascular form of the disease, it has been shown to be effective in only a subpopulation of patients (Nagai et al., 2016). Importantly, no treatment options are available for the early and intermediate stages of AMD. The lack of treatments is in part due to the complexity of the disease, as not only multiple genetic and environmental risk factors but also different cell types within the inner and outer retina, have been shown to be involved in the pathophysiology of AMD (Malek and Lad, 2014; Malek et al., 2018; Choudhary and Malek, 2020). Therefore, it is vital to further understand the molecular mechanisms underlying disease development and progression, in concert with the temporal development of pathological changes that occur in the retina. This is necessary in order to identify potential therapeutic targets. Herein, we will review the pathology and visual deficits associated with the different clinical subtypes of AMD and outline the pathogenic pathways linked to the development of AMD, with a focus on the growing body of evidence indicating that stress and injury to AMD vulnerable cells including photoreceptors, retinal pigment epithelial cells (RPE), retinal immune cells and choroidal endothelial cells, is a crucial component of the disease.

## AMD Classification and Grading

**FIGURE 1:**
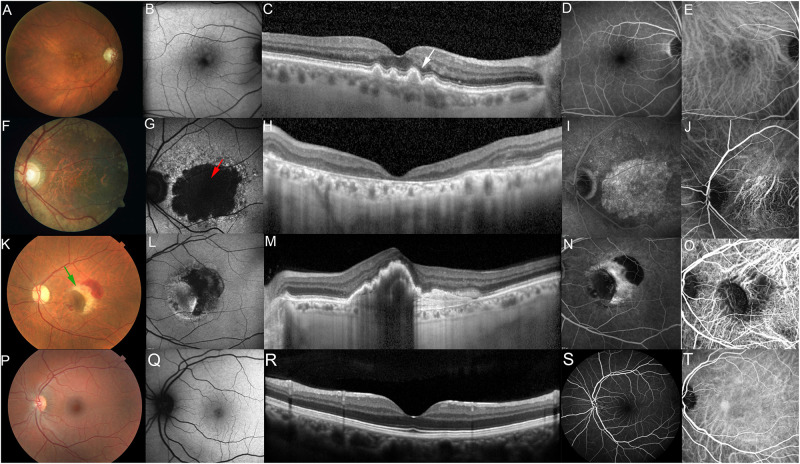
Photomicrographs showing different stages of AMD. Photomicrographs showing different stages of AMD compared to normal macula. Non-exudative AMD, intermediate (**A–E**, fundus photo, fundus autofluorescence, optical coherence tomography, fundus angiography and indocyanine green, respectively) showing drusen (white arrow), non-exudative AMD, advanced with subfoveal involvement **(F–J)** showing large, central GA (red arrow), Exudative AMD **(K–O)** showing Choroidal Neovascular Membrane (CNVM) and retinal hemorrhage (green arrow), Normal macula **(P–T)**.

The hallmark lesions of the *early stages of dry AMD*, often referred to as non-neovascular or non-exudative AMD (Figure 1), are sub-RPE deposits called drusen, derived from the German word for node or geode. Drusen formation have been noted in the peripheral regions with age, however, in early dry AMD they become larger and are found within the macula. Other indicators of dry AMD are RPE abnormalities, hyperpigmentation, and atrophy, as well as choriocapillary loss (Mullins et al., 2011; Malek and Lad, 2014) distinctive morphology from that seen in the normal posterior pole (Figures 1P–T). Clinically, drusen are small, yellowish appearing lesions located between the basal lamina of the RPE and the inner collagenous layer of Bruch’s membrane (a penta-laminar extracellular matrix, upon which the RPE cells reside) (Figures 1A–E). Histological evaluation of AMD donor tissue along with *in vivo* imaging of the posterior pole of AMD patients using optical coherence tomography (OCT) has revealed the presence of a variety of deposits characteristic of aging and AMD, beyond drusen, including basal laminar deposits, present between the RPE and its basal lamina, containing lipid-rich material and wide-spaced collagen; basal linear deposits containing phospholipids and located between the RPE basal lamina and Bruch membrane, within the same plane as drusen; and sub-retinal drusenoid deposits containing some established drusen markers (e.g., unesterified cholesterol, apolipoprotein E and complement factor H), but not all (e.g., esterified cholesterol) (Rudolf et al., 2008; Chen et al., 2020). Drusen size has been instrumental in classifying the severity of disease with small drusen, sized up to 63 μm in diameter; intermediate, sized between 64 and 125 μm in diameter; and large drusen exceeding 125 μm in diameter. These deposits have further been categorized based on their shape and boundaries, referred to as hard when they present with well-demarcated borders, soft with poorly demarcated borders and confluent when drusen are continuous without clear borders. In general, an eye with large, soft, and confluent drusen is at a higher risk of progressing to either of the advanced forms of AMD, geographic atrophy or choroidal neovascularization, relative to an eye with only hard drusen.

The *non-neovascular advanced stage of dry atrophic AMD* also known as geographic atrophy involves degeneration of the RPE, retina and the choriocapillaris with well-demarcated borders, resembling the map of a ‘continent’ (Figures 1F–J). The atrophic regions tend to be multi-focal, may or may not involve the foveal center (Ferris et al., 2013; Spaide et al., 2018) and often present bilaterally (Mann et al., 2011). The *wet or neovascular advanced form of AMD* is characterized by the presence of vascular growth from the choroid penetrating Bruch’s membrane, referred to as choroidal neovascularization, within the macula (Figures 1K–O). Though wet AMD is less frequent than dry AMD, the need for successful therapies is paramount as it is responsible for 90% of acute blindness. The clinical manifestations of neovascular AMD are varied and include subretinal and intraretinal fluid, retinal, subretinal, or sub-RPE hemorrhage, lipid exudates, plaque-like yellow-green choroidal neovascular membranes, RPE detachment, and RPE tear. In the end-stages, the neovascular membrane may evolve into a ‘disciform scar’ (hypertrophic, fibrovascular, or atrophic macular scar) causing permanent central vision loss (Ferris et al., 2013; Spaide et al., 2018).

It is important to note that geographic atrophy and choroidal neovascularization are not mutually exclusive as the atrophic retina may result in the development of a neovascular lesion (mostly at the edge of the atrophic region, especially if the contralateral eye is wet), and wet AMD may proceed to macular atrophy.

## Epidemiology and Risk Factors of AMD

The complexity of AMD lies not only in the variety of pathologies associated with the disease, but it is also reflected in the number of risk factors identified to date. Formative population-based investigations and genome-wide association studies, have revealed significant knowledge about AMD prevalence and genetic risk, respectively. The landmark study from 1992, “The Beaver Dam Study,” provided one of the first estimates of the prevalence of features of maculopathy including soft drusen, pigmentary abnormalities, choroidal neovascularization, and geographic atrophy, over a broad spectrum of ages (Klein et al., 1992). In general, the prevalence of advanced forms of the disease (wet AMD and geographic atrophy) was discovered to increase with each decade of life, being the highest after 75 years of age (Mitchell et al., 1995; Vingerling et al., 1995; Congdon et al., 2004; Joachim et al., 2015). The higher frequency of more severe macular pathology in the elderly, especially in the aging western population, brought to light the severity of this disease as an ongoing public health problem. Epidemiologic studies have also identified key risk factors for AMD, with advanced age acknowledged as the main one and cigarette smoking coming in second. Additional risk factors include but are not limited to positive family history, sex (female), hyperopia, light iris color, hypertension, hypercholesterolemia, cardiovascular diseases, obesity, and elevated inflammatory markers (Seddon et al., 1996, 2003; Age-Related Eye Disease Study Research Group, 2000; Hyman et al., 2000; Smith et al., 2001; Klein et al., 2003; Tomany et al., 2004; Malek and Lad, 2014; Armstrong and Mousavi, 2015). Importantly, the prevalence of the advanced forms of AMD appears to vary in different ethnic and racial groups, with the highest risk reported in the Caucasian population (5.4%) and lowest in African-Americans (2.4%); and the risk for Hispanics and Asians falling in between (4.2 and 4.6%, respectively) (Frank et al., 2000; Klein et al., 2004; Choudhury et al., 2016; Cheung et al., 2017).

Large genome-wide association studies of AMD, to date, have identified 52 genetic variants at 34 genetic loci associated with AMD. These genes harbor mutations that affect various biological pathways, including complement regulation, lipid metabolism, extracellular collagen matrix, angiogenesis, and all-*trans*-retinaldehyde metabolism, to name a few. Two major susceptibility genes for AMD that have been the focus of intense investigation, are the well-characterized CFH (1q31) that codes complement factor H, and poorly understood ARMS2 (10q26) (Hageman et al., 2005; Jakobsdottir et al., 2005; Klein et al., 2005; Scholl et al., 2005; Souied et al., 2005). The CFH mutation confers a 4.6- and 7.6-fold increased risk for AMD, while the ARMS2 mutation confers a 2.7- and 8.2-fold in heterozygotes and homozygotes, respectively. Most recently family-based exome sequencing studies have identified rare coding variants for novel candidate genes at eight previously reported loci, with 13 additional candidates detected outside of known regions, further highlighting the multi-factorial nature of AMD (Fritsche et al., 2013; Cheng et al., 2015; Fritsche et al., 2016; Gorin et al., 2016; Han et al., 2020). Genetic testing is currently available for AMD, but it is controversial and not officially recommended, given the limited treatment options available to patients. However, with the rapid advancements in this research field, this is likely to change soon (Edwards, 2006; Chew et al., 2015; Stone, 2015; Cascella et al., 2018; Warwick and Lotery, 2018).

## AMD-Driving Pathogenic Pathways

Despite extensive research, we still do not fully understand critical drivers involved in the initiation of AMD and progression from the early to advanced stages. This, in turn, has made predicting progression and effective treatments difficult. However, breakthroughs in identifying probable pathogenic pathways and molecular mechanisms associated with disease, born out of a consolidation of AMD pathologies, identified through observations of *in vivo* and *ex vivo* tissues, epidemiological studies, and in particular high-risk genes linked with AMD development, have been instrumental in the pursuit of animal models and potential therapies. These pathways, which are also often related to aging, include but are not limited to: complement activation, lipid trafficking and metabolism, vitamin A cycle/metabolism, proteostasis, bioenergetics, autophagy/mitophagy, extracellular matrix turnover, choroidal vascular dropout, and last but not least oxidant-induced and non-oxidant associated cellular injury and stress (Pool et al., 2020). The possible roles of each of these pathways in AMD warrant a special review in and of themselves. However, the rest of this review will focus on the impact of various stress modalities on cells vulnerable in AMD, whose induction has been attributed to modifiable dietary and environmental factors as well as factors that remain unknown.

## Impact of Oxidant and Non-Oxidant Stress and Injury on AMD

**FIGURE 2:**
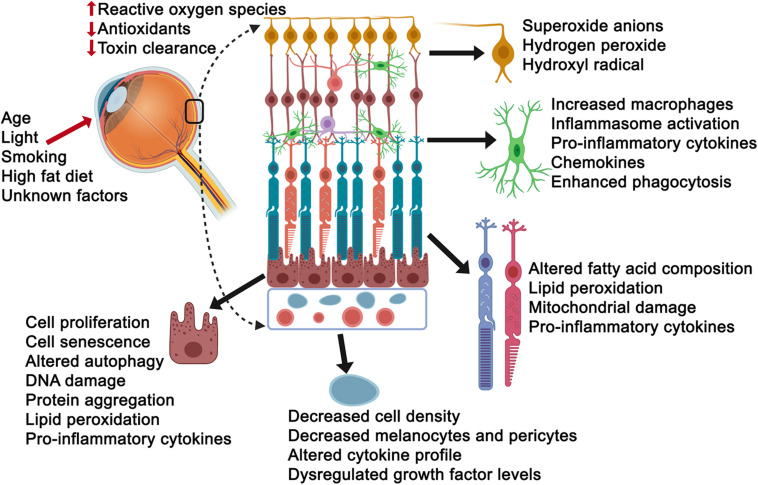
Consequences of stress exposure on retinal cells. Age, light exposure, smoking, high fat diet and unknown factors contribute to elevated reactive oxygen species, decreased intracellular anti-oxidant levels and toxin clearance mechanisms of retinal cells including the ganglion cells (yellow), microglial cells (green), photoreceptors (aqua/orange or light purple/red), retinal pigment epithelial cells (burgundy) and choriocapillaris/endothelial cells (light blue). Select consequences have been listed next to each cell.

Oxidative stress is often defined as a disturbance in the equilibrium between the amounts of reactive oxygen species and antioxidant production/detoxification capacity of cells. This equilibrium is critical for cell and tissue survival such that the consequence of any imbalance would be tissue injury. Injury to cells, however, can also occur in response to environmental factors and aging in general, compromising the tissues ability to respond effectively and counter stress (Luo et al., 2020). Importantly, the cells response to injury can also vary in accordance with the level of stress (low versus high) and the length of exposure (acute versus chronic), such that young healthy cells may counter acute stress more formidably that aged cells, specifically effecting cellular processes including autophagy, phagocytosis, proteosomal degradation, toxic clearance and metabolism, among others. Thus, it is not surprising that stress also has a major impact on aging neurodegenerative diseases such as AMD. Beyond the age factor, the retina is particularly vulnerable to photo-oxidative stress as it is chronically exposed to light (B Domènech and Marfany, 2020). Visual transduction pathways can result in reactive oxygen species production in response to oxidation of the building blocks of the photoreceptor outer segments, polyunsaturated fatty acids. Other mechanisms that put the retina in the line of fire for vulnerability to stress include modifiable behavioral risks, including smoking and indulging in diets rich in high fat and cholesterol (Klettner et al., 2013). Additional evidence for stress comes from proteomic studies of Bruch’s membrane tissue from AMD donors, revealing the presence of oxidative products (Beattie et al., 2010; Yuan et al., 2010), and the AREDS studies, which have shown an association between reduced prevalence of AMD and high dietary intake of antioxidants (Chew et al., 2013, 2014). With all this in mind, it is not surprising that there is a large body of evidence pointing to oxidant and non-oxidant stress as a bona fide pathobiologial process in AMD, including *in vitro* and *in vivo* studies examining AMD-vulnerable cells and tissues, which we will further review below (Figure 2).

### Retinal Ganglion Cells

The innermost retinal layer is primarily composed of ganglion cells. Though this layer is not a primary vulnerable site in AMD, thinning has been reported in dry AMD patients (Yenice et al., 2015) and as a consequence of retinal remodeling following photoreceptor degeneration (Garcia-Ayuso et al., 2018). Furthermore, a subset of ganglion cells contain melanopsin and are light-sensitive, significant given this layer is exposed to chronic light (Garcia-Ayuso et al., 2015). In response to overproduction of reactive oxygen species including superoxide anions, hydrogen peroxide, and hydroxyl radicals, ganglion cells die (Cao et al., 2015). However, they have also been shown to be remarkably resistant to cell death induction by these stressors in part due to their endogenous peroxides (Kortuem et al., 2000). As expected, this has led to a quest for neuroprotectives. *In vitro* and *in vivo* studies have revealed protective roles for master antioxidant defense regulators including the nuclear transcription factor kB (NF-kB) and nuclear factor – erythroid 2 – related factor 2 (NRF2) as well as polysaccharides, growth factors, including transforming growth factor beta, nerve growth factor, and brain-derived neurotrophic factor; endogenous antioxidant factors including glutathione, superoxide dismutase, and catalase to name a few (Pietrucha-Dutczak et al., 2018).

### Microglia

The retinal microglia are resident immune cells thought to be critical to the initiation of retinal inflammation (Rashid et al., 2019). A major consequence of oxidative and non-oxidant stress is inflammation. Though traditionally, abnormal microglial activity has been associated with retinal diseases including diabetic retinopathy, hereditary retinopathies, and glaucoma, recent evidence points to a role in AMD as well (Fletcher, 2020). Enlarged amoeboid microglia have been found adjacent to RPE cells overlying drusen in AMD retinal sections and may be a potential source for NLRP3 (NOD-, LRR- and pyrin domain-containing protein 3) inflammasome activation of RPE cells and increased pro-inflammatory cytokines such as IL-1 beta (Madeira et al., 2015). *In vitro* culture studies have shown that conditioned media from reactive microglial cells can trigger caspase-mediated photoreceptor cell death (Madeira et al., 2018). *In vivo* studies have also provided evidence for a role of microglia in retinal phenotypes associated with AMD. For example, in mice exposed to bright white, photoreceptor cell death, and retinal degeneration occurs along with migration of microglial cells to the outer retina (Wang et al., 2014). Another mouse model that presents with dry AMD phenotypes involves immunizing mice with carboxyethylpyrrole-adducted proteins. In these mice there is evidence of infiltrating phagocytes around degenerating photoreceptors and RPE cells (Hollyfield et al., 2008; Hollyfield, 2010). The question as to whether or not infiltrating macrophages are beneficial or detrimental is a complex one and ties into why the cells accumulate to begin with, which may be due to an increase in migration of monocytes into the retina or failure of immune cell clearance. In the carboxyethylpyrrole immunized mouse model, though sub-retinal macrophages are present, they are not seen in areas with severe RPE degeneration, suggesting that they may have a beneficial effect, perhaps in removing debris. Importantly in the wet laser-induced experimental mouse model of AMD, recruitment of ameboid microglia and mononuclear phagocytes are seen within the neovascular lesion, the number of which varies with the severity and nature of the lesion (fibrotic versus leaking), reflecting the dynamic nature of these cells (Crespo-Garcia et al., 2015; Zhou et al., 2017). As a consequence of microglial recruitment to the retina, there is enhanced phagocytosis and production of pro-inflammatory factors. This, in turn, can impact the integrity of the neural retina resulting in thinning of the outer nuclear layer (Karlstetter et al., 2015; Zhao et al., 2015).

The effect of oxidative stress on retinal microglia in AMD is still a relatively new area of research, the mechanism of which is unknown. Though much may be extrapolated regarding microglial cells from other retinal diseases such as glaucoma, in which the effect of adenosine blockade, a neuromodulator which works through its receptor A_2__*A*_R present on microglial cells, has been investigated (Santiago et al., 2014). Blockade of the receptor has been associated with decreased reactive oxygen species levels and morphological changes in microglial cells associated with pressure changes including retinal degeneration. Studies using minocycline in light-induced retinal damage models and their impact on microglial cells have varied showing both a decrease in immunolabeling of CD11b of microglial cells, which may protect against loss of photoreceptors through inhibition of retinal microglia activation, and a delay in photoreceptor cell death, which was independent of a reduction in retinal microglial cells, following depletion of microglia cells using liposomal chlodronate (Yang et al., 2009; Peng et al., 2014; Ferrer-Martin et al., 2015). *In vitro* studies also support this hypothesis, in which conditioned media from activated microglia cells induce apoptosis in the 661W transformed photoreceptor cell line (Roque et al., 1999). Hypoxia can cause retinal microglial cells to produce IL-1 beta and TNF alpha, and this has been associated with retinal microvascular degeneration by inducing semaphorin 3A in neurons (Rivera et al., 2013). Finally, in an *ex vivo* retinal culture model for oxidative injury induced by hydrogen peroxide, a dose-dependent increase in microglia and elevation of CD11b expression was observed followed oxidative stress induction, with a time-dependent increase in IL-1 beta, iNOS, HSP70 at day 3 and TNF alpha and IL-1 beta at day 8 (Hurst et al., 2017). Therapeutically, hypothermia used to counter hypoxia as a potential therapy for retinal degenerations has been shown to protect microglia numbers as well as CD11b expression (Maliha et al., 2019). Finally, in a mouse model of retinitis pigmentosa, MutY homolog-mediated (MUYTH-mediated) base excision repair (BER) in oxidative microglial activation has been proposed to be a novel target to dampen disease progression, able to suppress microglial activation and photoreceptor cell death (Nakatake et al., 2016).

### Photoreceptors

The major light-sensing neurons in the retina are the rod and cone photoreceptors, vulnerable in AMD in part due to their high metabolic demand. As mentioned earlier given the degree of photo-oxidative stress photoreceptors are exposed to, including light pollution by artificial light originating from commonly used technologies, a sundry of studies have been devoted to understanding the pathways impacted by oxidative stress. Using hypoxia as an inducer of stress, mice carrying the retinal degeneration 8 mutation, presented with accelerated photoreceptor degeneration and rosette formation, thinning of the central retina, and increased NADPH oxidase 4 in the outer nuclear layer (Lajko et al., 2017). Cool white light exposure (200 lux light-emitting diode) in mice has been shown to lead to photoreceptor cell death and alterations in fatty acid composition, specifically a decrease in docosahexanoic acid levels concomitant with an increase in stearic acid (Benedetto and Contin, 2019). In 661W cells, murine photoreceptor-like cells, knockdown of Nrf2 resulted in an increase in reactive oxygen species levels suggesting Nrf2 is again a key endogenous protective factor (Chen W. J. et al., 2017). Therapeutically, edaravone, a free radical scavenger, has been tested in a mouse model exposed to *N*-methyl-*N*-nitrosourea and found to inhibit outer nuclear layer thinning, cell death and oxidative stress markers (Tsuruma et al., 2012). Other treatments that have been tested include celastrol, a naturally occurring quinone methide triterpene, which demonstrated photoreceptor cell death suppression in BALB/c mice exposed to bright white light (Bian et al., 2016) and overexpression of cytochrome b5 in the *Drosophila melanogaster*, which resulted in suppression of blue light-induced retinal degeneration and lipid peroxidation (Chen X. et al., 2017). Importantly, it should be noted that age-related macular degeneration does not affect one cell type and when considering therapy the complex tissue should be studied. Indeed overexpression of catalase, an antioxidant, in RPE cells has been shown to protect its neighboring cells, the photoreceptors, from light damage, resulting in reduced 4 hydroxynonenal and nitrotyrosine levels, two markers of oxidative stress (Rex et al., 2004).

### Retinal Pigment Epithelium (RPE)

The retinal pigment epithelium (RPE) cells are hexagonal, polarized epithelial cells in close contact with photoreceptor outer segments at their apical side and Bruch’s membrane along their basal side. These highly specialized cells have many vital functions essential to retinal health, including daily phagocytosis and degradation of photoreceptor outer segments, light absorption, vitamin A metabolism, and heat exchange (Strauss, 2016). Additionally, RPE cells maintain the outer blood-retinal barrier and provide selective entry and removal of oxygen, nutrients, and metabolites (Strauss, 2005). With these multiple and diverse functions, RPE cells help maintain the photoreceptors and choriocapillaris’ health and function, thus playing a significant part in AMD’s pathogenesis.

Prolific investigations in AMD have proposed that oxidative stress is a common consequence of multiple risk factors involved in its pathogenesis. Macular high oxygen demand makes this part of the retina particularly susceptible to disturbed oxygen homeostasis. Various aerobic metabolism pathways produce reactive oxygen species; however, the primary source of their production is the mitochondria (Mao et al., 2014). For example, the identification of polymorphisms in mitochondrial MTND2^∗^LHON4917G, NADH dehydrogenase subunits, and mitochondrial superoxide dismutase 2, suggests a role for oxidative stress in AMD’s pathogenesis (Kraja et al., 2019). The LOC387715 polymorphism additionally supports this statement (Tong et al., 2010; Yang et al., 2010). The sources of oxidative stress in RPE cells range from high oxygen tension attributed to its close proximity to the outer retinal blood supply, the choriocapillaris, to the accumulation of autofluorescent lipid-protein aggregates that occur with aging, called lipofuscin (Sparrow and Yamamoto, 2012). Upon exposure to oxidative stress, intracellularly, not only are RPE proteins, lipids and DNA damaged, but also the mitochondria. Similar to photoreceptors, the post-mitotic nature of RPE cells preclude the rapid clearance of damaged mitochondria through cell division (Cai et al., 2000; Plafker et al., 2012). Like photoreceptors and ganglion cells, the role of Nrf2 for protection against phototoxic stress in RPE cells has been examined with *in vitro* studies demonstrating that sulforaphane, an Nrf2 activator can protect RPE cells from blue light-induced damage (Gao and Talalay, 2004). Other antioxidants tested in RPE cells range from glutathione, which in its reduced form has been shown to be protective against tert-butylhydroperoxide induced injury of RPE cells, potentially directly reacting with photooxidized components of lipofuscin (Sternberg et al., 1993; Yoon et al., 2011); to vitamins and their analogs including alpha-tocopherol (vitamin E), ascorbic acid (vitamin C) and beta-carotene, a precursor of vitamin A (Kagan et al., 2012).

### Choriocapillaris

The choriocapillaris is the complex fenestrated capillary layer of the choroid providing oxygen and nutrients to the RPE/neural retina. It is located immediately adjacent to Bruch’s membrane. Recently, the importance of the integrity of the choriocapillaris in all three clinical sub-types of AMD has been brought to the light with seminal studies demonstrating its vulnerability in non-neovascular or dry AMD (Chirco et al., 2017). Studies of human donor tissue from dry AMD patients revealed a loss in the density of the choriocapillaries (choriocapillary dropout), represented as an increase in non-perfused capillary segments also known as ‘ghost vessels’ (Mullins et al., 2011), while OCTA studies indicate thinning of the choroid, concomitant with increased average choriocapillaris signal void size, compared to eyes without neovascular AMD (Choi et al., 2015). Interestingly, in flatmount analyses of the choroidal tissue from geographic atrophy patients, the choriocapillaris appears intact in some regions adjacent to RPE loss, suggesting vulnerability in these patients is initially at the level of the RPE and perhaps secondarily effecting the choriocapillaris (McLeod et al., 2009). Extensive choriocapillary loss is seen in neovascular AMD, even in regions where the RPE appears to be intact (Moreira-Neto et al., 2018).

The impact of stress on the choriocapillaris is a burgeoning area of research with few studies so far, some in which photo-oxidative stress has served as the measurable endpoint. Most have involved the use of *in vitro* cultures exposed to blue-light or oxidative stress inducers such as hydrogen peroxide. Others include light-induced lipid peroxides localized to the choroid in the choroidal endothelial cells and melanocytes of albino (BALB/cJ) mice. An additional *in vivo* study tested the effect of overexposure to green light induced oxidative stress in choroidal endothelial cells in albino mice, observing oxidative damage to DNA impacting melanotyes and pericytes. Interestingly light-induced photo-oxidative stress resulted in activation of the NF-κB signaling pathway, which has been shown to be in response to oxidative stress (Wu et al., 2005). These studies primarily use albino mice as photo-oxidative stress induction in pigmented mice has been difficult. Therapeutically, sirt 1 (silent information regulator 1), which is activated when changes in cellular redox state occur, has been proposed as a potential target. *In vitro* studies using a monkey choroidal endothelial cell line (RF6A) exposed to sirtuin inhibitors points to a significant increase in reactive oxygen species production (Balaiya et al., 2017). Translocator protein (TSPO), a cholesterol-binding protein involved in mitochondrial cholesterol transport has been found to be expressed in the mitochondria of choroidal endothelial cells (Biswas et al., 2018). When exposed to TSPO ligands, production of reactive oxygen species by choroidal endothelial cells are reduced and there is an increase in antioxidant capacity, and reduction of pro-inflammatory cytokines induced by oxidized low-density lipoproteins, suggesting TSPO may be a potential therapeutic means to reduce oxidative stress in the choroidal endothelial cells. Finally, tert-butylhydroperoxide (tBH) mediated oxidative stress reduces survival of choroidal endothelial cells *in vitro*, and RPE cells exposed to tBH-mediated oxidative stress secrete increasing amounts of bFGF but not vascular endothelial growth factor (VEGF) in culture and support proliferation of choroidal endothelial cells, suggesting a mechanism leading to neovascularization as seen in wet AMD (Eichler et al., 2008). In conditions in which there is elevated VEGF, choroidal endothelial cells produce increased levels of reactive oxygen species, which can be prevented by NADPH oxidase inhibitors, as confirmed in the laser-induced choroidal neovascularization model (Monaghan-Benson et al., 2010). Finally, a mouse model lacking the anti-oxidant enzyme CuZn superoxide disumutase have been reported to develop neovascular lesions (Imamura et al., 2006).

Pigment epithelial-derived factor (PEDF) expression has also been shown to impact the oxidative state of choroidal endothelial cells. PEDF is an endogenous inhibitor of angiogenesis. Choroidal endothelial cells isolated from PEDF knockout mice demonstrated heightened sensitivity to hydrogen peroxide challenge with an increase in apoptotic cells, oxidative stress, and pro-inflammatory cytokine profile, along with increased cellular proliferation, decreased adhesion and migration (Park et al., 2011). Polypoidal choroidal vasculopathy, a late stage of neovascular AMD, is characterized by abnormal branching in the vascular networks and the presence of polypoidal or aneurysmal dilations, with the choroidal vessels displaying hyalinization. These dilations have been suggested to be the result of alternations in elastin, homocysteine-associated oxidative stress, and endothelial dysfunction. Interestingly, pretreatment of RF/6A cells subjected to paraquat to induce oxidative stress, with fenofibrate, a peroxisome proliferator activated receptor (PPAR) alpha agonist, resulted in decreased cellular apoptosis, diminished changes in mitochondrial membrane potential, increased expression of peroxiredoxin, thioredoxin, Bcl-2 and Bcl-xl and reduced BAX, pointing to fenofibrates anti-oxidant properties, as a potential adjunct therapy (Hsu et al., 2020). The receptor TNF alpha R2 is expressed in choroidal vascular cells, RPE, and Mueller cells and it has been shown that TNF alpha contributes to choroidal neovascularization by upregulating VEGF through reactive oxygen species activation of the beta-catenin signaling pathway (Wang et al., 2016). The expression of thrombospondin-1, which is another endogenous inhibitor of angiogenesis and inflammation, has been shown to regulate choroidal endothelial cells. Interestingly thrombospondin 1 knock out in choroidal endothelial cells results in increased levels of thrombospondin 2, phosphorylated endothelial and inducible nitric oxide synthase, which are associated with significantly high levels of nitric oxide and oxidative stress (Fei et al., 2014). In addition to supplementation with carotenoids such as zeaxanthin and lutein, potential therapies targeting reactive oxygen species production in the choroid tested *in vitro* and *in vivo*, have been the use of resveratrol, which showed to inhibit proliferation of hypoxic choroidal endothelial cells in association with an increase in caspase 3, and may serve as a therapeutic option to be considered for targeting stress in choroidal neovascularization (Balaiya et al., 2013; Nagai et al., 2014).

## Successes and Failures of AMD Therapies and the Pipeline

Over two decades ago, a diagnosis of wet AMD was a dreadful one as no treatment options were available to patients. However, a breakthrough came when the Food and Drug Administration (FDA) approved the first anti-angiogenic drug, Macugen (Pegaptanib sodium injection, Eye Tech Pharmaceuticals, currently OSI Pharmaceuticals, Long Island, NY, United States), to be used in the treatment of wet AMD. Since then, the field has blossomed with an AMD disease prognosis changing to one in which therapeutic options leave more than 90% of patients maintaining their vision [losing <15 ETDRS (Early Treatment Diabetic Retinopathy Study) letters] after 1 year of treatment (Heier et al., 2012). In recent years, though Macugen showed promise in slowing down vision loss in patients with wet AMD it has quickly been replaced by more effective medications. Currently, the three most widely used drugs provide an anti-angiogenesis effect by blocking VEGF. Two are FDA approved, and one is being used off-label. The FDA approved ranibizumab (Lucentis, Genentech) in 2006, a recombinant humanized antibody fragment (Fab) that binds and inhibits all active forms of VEGF-A and their functional degradation products (Brown et al., 2006; Rosenfeld et al., 2006). Aflibercept (Regeneron) was approved by the FDA shortly after in 2011. It is a soluble protein that acts as a VEGF receptor decoy by combining ligand-binding elements of the extracellular domains of VEGFR1 and two fused to the constant region (Fc) of the immunoglobulin G (IgG). Because of its greater half-life, the drug can be used in a bimonthly regimen, significantly reducing the number of necessary intravitreal injections (Schmidt-Erfurth et al., 2014). Finally, an off-label drug for AMD treatment, Bevacizumab (Genentech), is a full-length humanized monoclonal antibody against VEGF, with a longer systemic half-life than other anti-VEGF agents (e.g., about 21 days for bevacizumab, vs. 2.2 h for ranibmizumab) (Ferrara et al., 2004; Wang et al., 2004; Yang and Wang, 2004; Heier et al., 2012; Bakall et al., 2013; Busbee et al., 2013; Rofagha et al., 2013; Ferrone et al., 2014; Grewal et al., 2014; Schmidt-Erfurth et al., 2014; Bhisitkul et al., 2015; Avery et al., 2017). It is important to note that increased oxidative stress plays an important role in AMD, triggering the expression of VEGF-A, in this case, believed to serve as a survival factor. It follows that anti-VEGF therapy may negatively impact cell survival under oxidant injury conditions, the extent to which can only be determined through a systematic study examining the impact of anti-VEGF on reactive oxygen species levels following oxidative stress.

Despite the availability of treatments for wet AMD patients, about a third of patients have visual decline by 15 letters or more (Moutray and Chakravarthy, 2011; Rofagha et al., 2013; Bhisitkul et al., 2015). Importantly, repeated intravitreal injections lead to significant socioeconomic burden (Brown et al., 2005). Although available therapies are grossly successful, wet AMD still remains the center of interest of leading pharmaceutical companies. Numerous new injectable medications are coming down the pipeline, only some of which we have space to review here (see also Table 1). Already approved by the FDA (October of 2019) is brolucizumab, developed by Novartis and Alcon Labs. This humanized single-chain antibody fragment that inhibits all isoforms of VEGF-A has already proven to achieve the clinical endpoint on the 12-week dosing interval following the induction (Dugel et al., 2020). The real-world experience to follow the clinical trial results is still mandated to make this medication more competitive. In 2019, a new anti-VEGF agent, conbercept by Lumitin (China), was approved locally to treat wet AMD and reported to be safe and efficient (Cãlugãru and Cãlugãru, 2019; Liu et al., 2019). From Roche/Genentech currently under investigation is a drug that simultaneously inhibits VEGF-A and angiopoietin-2, faricimab (Hussain et al., 2019). Aerpio is developing ARP-1536, a humanized monoclonal antibody that targets the extracellular domain of vascular endothelial protein tyrosine phosphatase (Al-Khersan et al., 2019). Opthea is developing OPT-302, a soluble form of human VEGF receptor-3 that blocks VEGF-C and VEGF-D to be used combined with an anti-VEGF-A agent (Al-Khersan et al., 2019). Kodiak Sciences is working on a novel, anti-VEGF antibody biopolymer conjugate to treat wet AMD (KSI-301). The first results on treatment naïve eyes with neovascular AMD are expected in 2020 (Al-Khersan et al., 2019). Regenxbio is developing a gene therapy, RGX-314, as a one-time subretinal injection. It consists of the NAV AAV8 vector encoding a VEGF inhibiting antibody fragment (Al-Khersan et al., 2019). Allergan is in Phase 3, successfully exploring a novel agent with designed ankyrin repeat proteins (DARPin) technology to be used as an intravitreal injection to inhibit all isoforms of anti-VEGF-A. Thus far, in *in vitro* experiments, the VEGF-A binding affinity of abicipar pegol was found to be similar to that of aflibercept and greater than that of ranibizumab and bevacizumab (Callanan et al., 2018; Rodrigues et al., 2018; Moisseiev and Loewenstein, 2020; Sharma et al., 2020). Regenxbio and Adverum Biotechnologies are developing gene therapies, RGX-314 and ADVM-022, respectively, as one-time subretinal injections. Regenxbio’s approach utilizes the NAV AAV8 vector encoding a VEGF inhibiting antibody fragment (Al-Khersan et al., 2019), while ADVM-022 is an AAV.7m8-aflibercept gene therapy product. PanOptica is going with a less invasive, topical application option (once-a-day drop) of pazopanib (PAN-90806), a molecule that blocks VEGF receptor 2 via tyrosine kinase inhibition (Hussain and Ciulla, 2017; Patra et al., 2018; Al-Khersan et al., 2019).

**TABLE 1:** Approved or advanced in trials Wet AMD treatments.

**Generic**	**Brand name**	**Manufacturer**	**Target**	**FDA approved/year**	**Phase in trials**
Pegabtanib	Macugen	OSI Pharmaceuticals	165 isoforms VEGF-A/Pegylated RNA aptamer	Yes/2004	Concluded

Ranimizumab	Lucentis	Genentech	All isoforms VEGF-A/Monoclonal anti-VEGF (Fab) fragment	Yes/2006	Concluded

Bevaclzumab	Avastin	Genentech	All isoforms VEGF-A/Monoclonal Ab	No	Concluded/used off-label

Aflibercept	Eylea	Regeneron	All isoforms of VEGF-A, VEGF-B, and PIGF15/Fusion protein: VEGFR-1,2 fused with lgG1 Fc	Yes/2011	Concluded

Brolucizumab	Beovu	Novartis/Alcon	VEGF-A.B, PIGF/Single-chain anti-VEGF Ab/fragments (scFv)	Yes/2019	Concluded

Conbercapl		Lumitin	All isoforms of VEGF-A, VEGF-B, VEGF-C, and PIGF32/Fusion protein: VEGFR-1,2 fused with lgG1 Fc	No/approved in China	Phase 3/NCT03577899

Faricimab		Genentech/Roch	All isoforms VEGF-A and Ang-2/Bispecific monoclonal Ab	No	Phase3/NCT03823287

ARP-1536		Aerpio	All isoforms of VEGF-A (inactivate), Tie2 (activate)/reactivating monoclonal antibody	No	Predinical development

OPT-302		Opthea	VEGF-C and VEGF-D/Trap’ molecule (VEGF-C and VEGF-D)	No	Phase2/NCT03345082

KSI-301		Kodiak Science	All isoforms of VEGF-A/anti-VEGF antibody biopolymer conjugate	No	Phase 1/NCT04049266

Abicipar pegol		Allergan	Ankyrin repeat proteins (DARPin)Zall isoforms of anti-VEGF A	No	Phase3/NCT02462928

RGX-314		Regenxbio	All isoforms of VEGF-A/NAV AAV8 vector containing a gene encoding for a monoclonal antibody fragment	No	Phase2/NCT03066258

ADVM-022		Adverum Biotechnologies, Inc.	All isoforms of VEGF-A/AAVJmS-aflibercept	No	Phase 1/NCT03748784

PAN-90S06		Panoptica	VEGFR2/small-molecule tyrosine kinase inhibitor	No	Phase2/NCT03479372

Unlike for wet AMD, to date, there are no approved treatments for dry AMD. The groundbreaking Age-Related Eye Disease Study (AREDS) initially conducted from 1992 to 2001 concluded that daily supplementation with high antioxidants levels and zinc might reduce the risk of progression in about 25% of patients. Recently conducted supplemental studies have revealed that some patients may experience up to 85% risk reduction, while others may encounter a threefold increased risk of progression while on supplementation, depending on their genetic make-up (Seddon et al., 2016; Vavvas et al., 2018). Significant effort has been made in dry AMD treatment research, and currently, there are ongoing, promising clinical trials (see also Table 2). There is an ongoing effort in China to transplant human embryonic stem cells derived from RPE into the subretinal space of patients with advanced dry AMD (NCT03046407). Additionally, the Bionic Vision system PRIMA (retinal prosthesis) is being developed by Pixium Vision (NCT03392324). Hemera Biosciences is investigating AAVCAGsCD59, an ocular gene therapy product that causes normal retinal cells to increase their expression of a soluble form of CD59. Conveniently, the compound can be injected in the physician’s office. This soluble recombinant version of CD59 is designed to inhibit the formation of the membrane attack complex, the terminal step of complement-mediated cell lysis, to protect retinal cells (NCT03144999). Allegro Ophthalmics is planning a phase III trial to evaluate the safety and exploratory efficacy of risuteganib (Luminate) on dry AMD (NCT03626636). Risuteganib regulates mitochondrial dysfunction and downregulates oxidative stress response in order to restore retinal homeostasis. Opthotech is evaluating avacincaptad pegol (Zimura, a novel complement C5 inhibitor) when intravitreally administered in subjects with geographic atrophy (NCT02686658). Regenerative Patch Technologies has initiated a clinical trial to assess the feasibility of delivery and safety of human embryonic stem cell-derived retinal pigment epithelial cells on a parylene membrane (CPCB-RPE1) in patients with advanced, dry AMD (NCT02590692). Some additional potential treatments for dry AMD, advanced in clinical trials, are listed below. Alkeus Pharmaceuticals, Inc. propose visual cycle modifications as a treatment option (ALK001-P3001, NCT03845582), via the use of a modified form of vitamin A that replaces natural vitamin A in the body, thus slowing the production of damaging vitamin A dimers, postulated to slow the accumulation of toxic end products and therefore slow the progression of AMD (2019d). Soliris (Alexon), Genentech, and Apellis Pharmaceuticals are successfully investigating the role of complement inhibition in slowing down the progression of dry AMD. Their products, Eculizumab, Lampalizumab, and Pegcetacopan (respectfully), are currently undergoing Phase 2 and 3 clinical trials (NCT00935883, NCT03972709, NCT02247531, and NCT0350054) (Yehoshua et al., 2014; Yaspan et al., 2017; Holz et al., 2018). Additionally, anti-inflammatory agents have also been proposed to slow down dry AMD advancement. Genentech/Roche proposes the use of FHTR2163 (Genentech/Roche), a new antibody delivered by intravitreal injection that inhibits HTRA1, a serine protease gene HTRA1 as a major risk factor for wet AMD [Phase 2/NCT03972709 (Dewan et al., 2006)]. A Phase 2 clinical trial conducted by Allergen (NCT02087085) is investigating the neuroprotective role of intravitreal brimonidine for geographic atrophy, administered by a delayed-delivery system implant (2020a). Finally, Jenssen Pharmaceuticals is assessing non-stem cell-based therapy with Palucorcel (CNTO-2476), which uses human umbilical cord tissue-derived cells (hUTC), while Astellas Pharma is assessing a stem cell-based approach using human embryonic stem cells (hESC) as cell-based approach therapies to treat advanced dry stages of AMD [Phase 2/NCT01226628 and NCT03046407 (Lund et al., 2007; Schwartz et al., 2015)].

**TABLE 2:** Advanced in trials Dry AMD treatments.

**Generic**	**Brand name**	**Manufacturer**	**Target**	**FDA approved/year**	**Phase in trials**
PRIM A FS-US		Pixium Vision	Bionic Vision	No	N/A/NCT03046407

Risuteganib	Luminate	Allegro Ophthalmic	Mitochondrial dysfunction (oxidative stress)/integrin inhibitor	No	Phase 2/NCT03626636

ALK001-P3001		Alkeus Pharmaceuticals, Inc.	Modified vitamin A decreases rate of toxic dimer formation	No	Phase 3/NCT038455B2

AAVCAGsCDSS		Hamera Biosciences	MAC inhibition via CD59/gene therapy	No	Phase1/NCT03144999

Eculizumab		Soliris, Alexon	A humanized monoclonal antibody derived from the murine anti-human C5 antibody	No	Phase2/NCT00935883

Lampalizumab		Genentech	Antigen-binding fragment (Fab) of a humanized monoclonal antibody that acts as a selective inhibitor of complement factor D	No	Phase 2/MCT03972709

Pegcetacoplan (APL-2)		Apellis Pharmaceuticals	Synthetic molecule that selectively inhibits C3, effectively downregulating all three complement pathways	No	Phase 2/NCT03500549

Avacincaptad pegol	Zirnura	Ophthotech/lveric	Complement factor- C5 inhibitor	No	Phase 3/NCT02686658

CPCB-RPE1		Regenerative Patch Tech.	Human Embryonic Stem RPEs/RPE transplantation	No	Phase 2/NCT02590692

Brimo DDS		Allergan	Brimonidine implant/neuroprotection	No	Phase 2/NCT0208708S

CNTO-2476		Janssen Pharmaceuticals	Biological/non-stem cell-based therapy with palucorcel (CNTO-2476), which uses human umbilical cord tissue-derived cells (hUTC)	No	Phase 2/NCT01226628

hESC MA09-hRPE		Astellas Pharma Inc.	biological/sub-retinal Transplantation of hESC Derived RPE (MA09-hRPE)	No	Phase 2/NCT01344993

		Chinese Academy of Sciences	Human Embryonic Stem RPEs/RPE transplantation	No	Phase 2/NCT03046407

FHTR2163		Genentech/Roche	Antibody delivered by intravitreal injection that inhibits a serine protease gene (HTRAI)	No	Phase 2/NCT03972709

## Conclusion

As presented above, significant research is being done to investigate new therapeutics for both dry and wet AMD. The most successful therapies so far address aspects of wet AMD, leaving a large gap to be filled with therapies for dry AMD. Unfortunately, a large number of potential medications have been tested for dry AMD and have failed. Currently more candidates are undergoing clinical trials, some targeting the impact of stress on mitochondria as well as inflammation, emphasizing the importance of these pathways in the pathogenesis of AMD. Nevertheless, the very nature of the complex etiology of AMD dictates that future therapeutic protocols, will require treatments directed to more than one aspect of the pathobiology of AMD, thus advocating for additional effort to be invested in a multi-targeted approach to AMD treatment.

## Author Contributions

MH and GM contributed to the conceptualization, writing, and editing of this review. Both authors contributed to the article and approved the submitted version.

## Conflict of Interest

The authors declare that the research was conducted in the absence of any commercial or financial relationships that could be construed as a potential conflict of interest.
